# The Impact of Open versus Closed Computer-Aided Design/Computer-Aided Manufacturing Systems on the Marginal Gap of Zirconia-Reinforced Lithium Silicate Single Crowns Evaluated by Scanning Electron Microscopy: A Comparative In Vitro Study

**DOI:** 10.3390/jfb15050130

**Published:** 2024-05-15

**Authors:** Asaf Shely, Joseph Nissan, Ofir Rosner, Eran Zenziper, Diva Lugassy, Khadija Abidulkrem, Gil Ben-Izhack

**Affiliations:** 1 Department of Oral Rehabilitation, The Maurice and Gabriela Goldschleger School of Dental Medicine, Sackler Faculty of Medicine, Tel Aviv University, Tel Aviv 6997801, Israel; asafshely@gmail.com (A.S.); nissandr@gmail.com (J.N.); rosnerop@yahoo.com (O.R.); eranzen@gmail.com (E.Z.); khadijaa@mail.tau.ac.il (K.A.); 2 Department of Orthodontics, The Maurice and Gabriela Goldschleger School of Dental Medicine, Sackler Faculty of Medicine, Tel Aviv University, Tel Aviv 6997801, Israel; diva.lugassy@gmail.com

**Keywords:** open system, closed system, CAD-CAM, Primescan, Trios 4, ZLS, marginal fit, marginal gap, marginal discrepancy, SEM

## Abstract

This study aimed to compare the impact of CAD/CAM closed systems and open systems on the marginal gap of monolithic zirconia-reinforced lithium silicate (ZLS) ceramic crowns, as both systems are used in everyday dentistry, both chair-side and laboratory. For the closed system, 20 plastic teeth were scanned by a Primescan intra-oral scanner (IOS), and for the open system, the same number of plastic teeth were scanned by Trios 4 IOS. For the closed system, CEREC software was used, and for the open system, EXOCAD software was used. All 40 ZLS crowns were grinded by the same four-axis machine and cemented with Temp-bond, followed by self-adhesive resin cement. For each type of cement, an evaluation of the marginal gap was conducted by scanning electron microscopy (SEM). Before comparisons between the groups, a Kolmogorov–Smirnov test was performed on the study variables showing a normal distribution (*p* > 0.05). Independent *T* tests (α = 0.05) and paired-sample *T* tests (α = 0.05) were used. The independent *T* test found no significant mean marginal gap differences in the zirconia-reinforced lithium silicate crowns bonded with Temp-bond and scanned by Primescan (28.09 μm ± 3.06) compared to Trios 4 (28.94 μm ± 3.30) (*p* = 0.401), and there was no significant mean marginal gap differences in zirconia-reinforced lithium silicate crowns bonded with self-adhesive resin cement (Gcem ONE) and scanned by Primescan (46.70 μm ± 3.80) compared to Trios 4 (47.79 μm ± 2.59) (*p* = 0.295). Paired-sample *T* tests showed significantly higher mean marginal gaps with Gcem ONE compared to Temp-bond for the total mean marginal gap when scanning with Primescan (*p* = 0.0005) or Trios 4 (*p* = 0.0005). In everyday dentistry, both closed systems (Primescan with Cerec) and open systems (Trios 4 with Exocad) can be used to achieve an acceptable (<120 µm) marginal gap for ZLS CELTRA^®^ DUO single crowns. There is a significant difference between cementation with Temp-bond and Gcem ONE self-adhesive resin cement (*p* < 0.05).

## 1. Introduction

In the last few decades, the development of computer-aided design and computer-aided manufacturing (CAD/CAM) techniques has put the spotlight on the need for intra-oral scanners (IOSs) in prosthetic dentistry [1]. Many studies comparing between conventional and digital impressions have been carried out and have shown that the three-dimensional geometric scanning data (mesh cad) of single-unit crowns’ preparation obtained by IOSs is as accurate as that obtained by conventional ones; therefore, they are clinically acceptable [2,3,4,5].

PrimeScan^®^ (CEREC^®^ Primescan; Dentsply Sirona, Milford, DE, USA) and Trios 4^®^ (3Shape^®^ Trios 4, Copenhagen, Denmark) are IOSs that provide clinically acceptable results in terms of marginal gaps [6,7,8,9,10]. Both IOSs work with the confocal microscopy principle, which was patented by Minsky in the 1950s, and after overcoming many challenges over the decades, Minsky’s prototype had been evolved into a standard model known as confocal laser scanning microscopy (CLSM) [11].

CLSM is an optical technology that depends on a process called optical sectioning, by which, it obtains high-resolution images from a certain selected depth. An objective lens in CLMS focuses the laser beam, which passes through an aperture into a focal volume. The light, whose source is not the focal point, is suppressed by limited detector aperture, thereby blocking unsound light [12].

The above-mentioned working mechanism produces in-focus images. Two-dimensional images of a specimen are accurately obtained point-by-point by this system and reconstructed to be a final single three-dimensional picture by a computer [12]. The scans acquired by CLMS are of a high contrast and resolution degree due to the low scan speed and an optimum pinhole diameter, which enables the production of images with adequate signal-to-noise ratios (SNRs) [12,13].

The marginal gap is one of the critical criteria in determining the strength and long-term success of crowns and fixed dental prostheses (FDPs) and is defined as the distance from the internal surface of the dental restoration to the finish line of the teeth preparation [14]. A maximum marginal gap within 120 μm was reported by McLean and von Fraunhofer [15], and it is known that there are some factors which can affect the marginal gap; some of them are the milling or grinding systems (number of axis), types of IOS, measurement methods, location and type of finish line, type of restoration material and preparation design [16,17]. More recent studies suggested that an acceptable marginal gap should be below 100 μm [18,19,20], while some studies suggested that the maximum acceptable marginal gap can range between 75 and 160 μm [21,22]. A poor-fitting restoration may cause microleakage and bacterial colonization that can damage the abutment tooth and its periodontal tissues [23,24].

CELTRA^®^ DUO (Dentsply Sirona, Milford, DE, USA) is a zirconia-reinforced lithium silicate CAD/CAM material, also known as ZLS, which is a relatively new ceramic material. It has a composition which is dual microstructure: a homogeneous glassy matrix of lithium metasilicate (Li_2_SO_3_) and lithium orthophosphates (Li_3_PO_4_) with 10% added zirconium dioxide (ZrO_2_) which improves its mechanical characteristics. The material can be used for single partial and full restorations for both anterior and posterior regions due to high translucency and high biaxial flexural strength values [25,26,27]. In a recent study, a radial spacer of 90 microns was presented as the optimum spacer for CELTRA^®^ DUO [28].

The use of optical microscopes in the dental field, both for clinical use and research aims, is well known. A wide range of microscopy techniques are known; scanning electron microscopy (SEM) is one of them, with articles being published since 1962. SEM is being used in a variety of fields in dentistry: oral tissues, biomaterials, dental restorative materials, surface characteristics, fracture analysis and integrity of interfaces. In addition, SEM shows high resolution and high magnification (×50 to ×5000) and is based on non-destructive and non-invasive evaluation methods [29].

In everyday dentistry, there are two main CAD/CAM systems which dentists choose to use at the clinic: closes systems and open systems. When using the closed system, the dentist scans with an intra-oral scanner and designs and grinds or mills the restoration in house; this flow chart is usually restricted to one manufacturer (like Sirona Dentsply). The second is an open system where the dentist scans with any intra-oral scanner and then sends the Standard Tessellation Language (STL) file to the laboratory; the technician imports the file into a CAM system and designs and mills the restoration [30,31]. In the current literature, there are many studies with different measuring methods and materials which examined the marginal gap of single restorations manufactured by open systems or closed systems, with both showing acceptable marginal discrepancies between 51 µm to 90 µm [2,14,17,29].

After a comprehensive review of the literature, only a few studies comparing closed and open systems were found, but none compared the impact of a closed system versus an open system on the marginal gap adaptation of zirconia-reinforced lithium disilicate (CELTRA^®^ DUO) single crowns with PrimeScan^®^ (CEREC^®^ Primescan; Dentsply Sirona, Milford, DE, USA) and Trios 4^®^ (3Shape^®^ Trios 4, Copenhagen, Denmark). The influence of open and closed systems on the marginal gap, especially with current advanced IOS and CAD/CAM systems, needs more investigations. The first null hypothesis was that no difference would be found between open systems and closed systems regarding the marginal gap; the second null hypothesis was that no difference would be found between two different types of cements (temporary and permanent) regarding the marginal gap.

## 2. Materials and Methods

**Figure 1:**
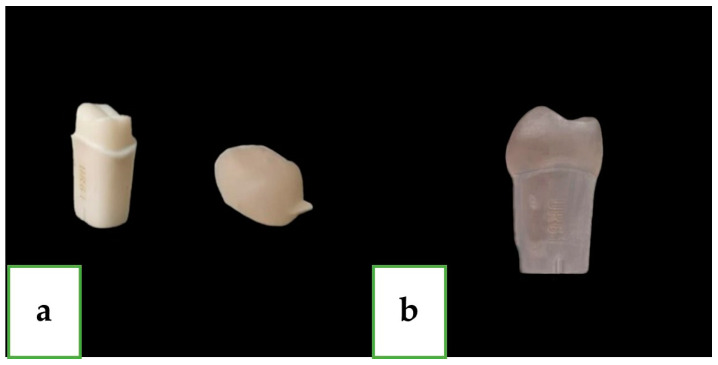
(**a**) Plastic abutment of first maxillary molar with crown (zirconia-reinforced lithium silicate). (**b**) Abutment and crown after gold coating.

In this research, 40 plastic teeth (20 for each group) were used; all teeth were identical maxillary first molars (FLUX 8634; Columbia Dentoform, Lancaster, PA, USA) with a uniform preparation which was made by the company with a machine. All teeth were made as abutments for single full ceramic crowns with identical parameters: a 1.2 mm shoulder finish line all around, an axial convergence angle of 6 degrees for all surfaces, and occlusal reduction of 2 mm. (Figure 1).

For the closed system group, half of the abutments (20) were scanned with IOS Primescan (CEREC^®^ Primescan; Dentsply Sirona, Charlotte, NC, USA); a virtual model was created (CEREC^®^ SW 5.2.4; Dentsply Sirona), and a well-experienced single user marked the finish line (A.S., 11 years of experience). The restoration of a single crown was designed (parameters were defined: occlusal contact strength of 25 µm, proximal contact strength of 25 µm, radial minimal thickness of 1000 µm, dynamic contact strength of 25 µm, occlusal minimal thickness of 1500 µm, margin thickness of 50 µm, a margin ramp width of 50 µm, a margin ramp angle of 60°, a radial spacer of 90 µm and an occlusal spacer of 120 µm) and grinded by a 4-axis grinding machine (CEREC MC XL^®^; Dentsply Sirona) from an ingot of zirconia-reinforced lithium silicate (CELTRA^®^ DUO, Sirona Dentsply, Milford, DE, USA).

For the open system group, the other half of the abutments (20) were scanned with IOS Trios 4 (3Shape^®^ Trios 4, Copenhagen, Denmark), and 20 STL files were produced and imported to an open CAD system (EXOCAD^®^ DentalCAD 2.2 Vallenta, Darmstadt, Germany). A virtual model was created, and a well-experienced single user marked the finish line (A.S., 11 years of experience). The restoration of a single crown was designed (same parameters were defined as described before) and grinded by the same 4-axis grinding machine (CEREC MC XL^®^; Dentsply Sirona) from an ingot of zirconia-reinforced lithium silicate (CELTRA^®^ DUO, Sirona Dentsply, Milford, DE, USA).

For the creation of a control group, all 40 crowns were cemented to the plastic abutments with Temp-bond (Temp-Bond™ NE™ Unidose; KaVo Kerr, Brea, CA, USA); during the cementation process, LUTRON ELECTRONIC ENTERPRISE CO. LTD FG-20KG was used to apply a constant and same pressure of 50 N·cm on each crown until the setting time passed following the recommendations of the manufacturer.

**Figure 2:**
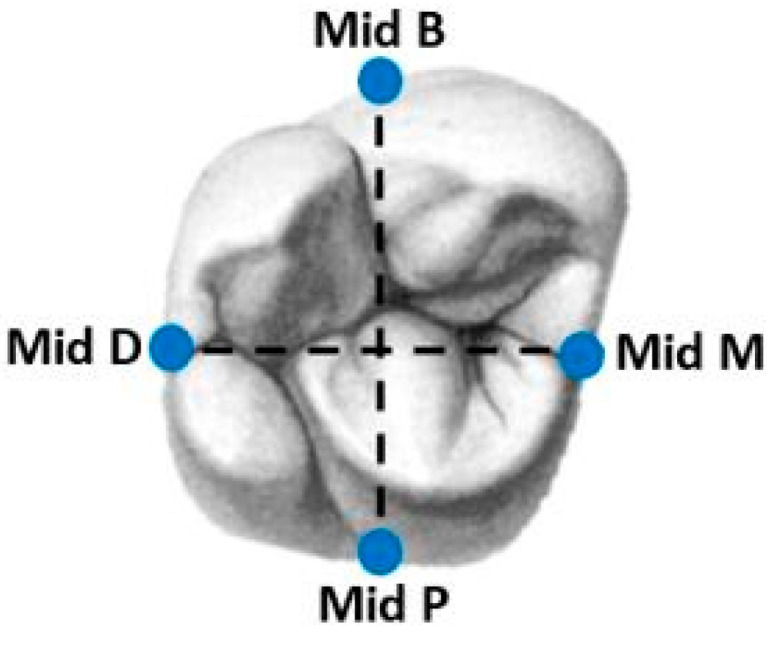
Four repeatable reference points for measurements.

The crown and abutment together were defined as a unit; for each unit, a mark was drilled on each surface (repeatable reference point) with a tungsten bur (FG330; Strauss&Co, Ra’anana, Israel). The four measuring sites were on the buccal, mesial, palatal and distal surfaces (Figure 2). Measurements were taken under a scanning electron microscope (SEM), and for these scans, a further process was carried out prior to scanning: a mini sputter (SC7620; Quorum, Laughton, East Sussex, England) was used to apply a uniform gold coating on all surfaces of the units (Figure 1).

**Figure 3:**
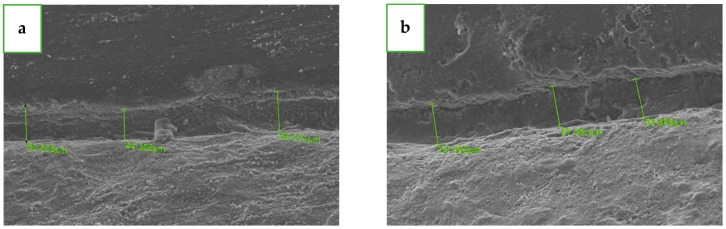
A view from the side of the measurement taken under SEM. The circumferential marginal gap (CMG) is the average of the three green lines. (**a**) Primescan group with Gcem ONE. (**b**) Trios group with Gcem ONE.

The same operator (K.A.) scanned all 40 units by SEM (JSM-IT100; JEOL, Akishima, Tokyo, Japan) and measured them using operation software (InTouchScope) at a magnification of ×250 in four locations (B, M, P and D, at the repeatable reference point) in the vertical dimension between the margins of the abutments and the crown. Three measurements were taken at each location (Figure 3), twelve measurements for each unit. The average of the three measurements at each location was defined as the circumferential marginal gap (CMG). For each group (Primescan and Trios 4), there were 20 CMGs at each location (B, M, P, D) and the average of these 20 measurements was defined as the mean marginal gap (MMG). For each group (Primescan and Trios 4), there were 4 MMGs at each location (B, M, P, D), and the average of these 4 measurements was defined as the total mean marginal gap (TMMG).

After finishing all measurements for the Temp-bond group, all 40 crowns were removed from the abutments, Temp-bond was thoroughly cleaned by a steamer (Orix, Tel-Aviv, Israel), and the cementation process with Gcem ONE Auto mix (GCA; GC, Alsip, IL, USA) was applied as described before. The same rigorous measuring methods were used with all 40 units as described before and by the same operator (K.A.).

A Statistical Package for Social Sciences for Windows Release 23.0 (SPSS Inc., Chicago, IL, USA) was used to perform the statistical analysis.

Before comparisons, the Kolmogorov–Smirnov normality test was performed on the study variables indicating normal distribution (*p* > 0.05). Due to the normality tests results, the independent *T* test (α = 0.05) was used for the comparison within groups (Temp-bond and Gcem ONE) and a paired-sample *T* test (α = 0.05) was used for the comparison between groups (Primescan and Trios 4). A sensitivity power analysis using G*Power showed that the dependent *T* test and independent *T* test with two groups, both *n* = 20, would be sensitive to the effect of Cohen’s f = 0.66 and 0.90, respectively, with 80% power (α = 0.05, two tail). The statistical significance level for this work is *p* < 0.05.

## 3. Results

A Kolmogorov–Smirnov test performed on the study variables indicated a normal distribution (*p* > 0.05).

**Figure 4:**
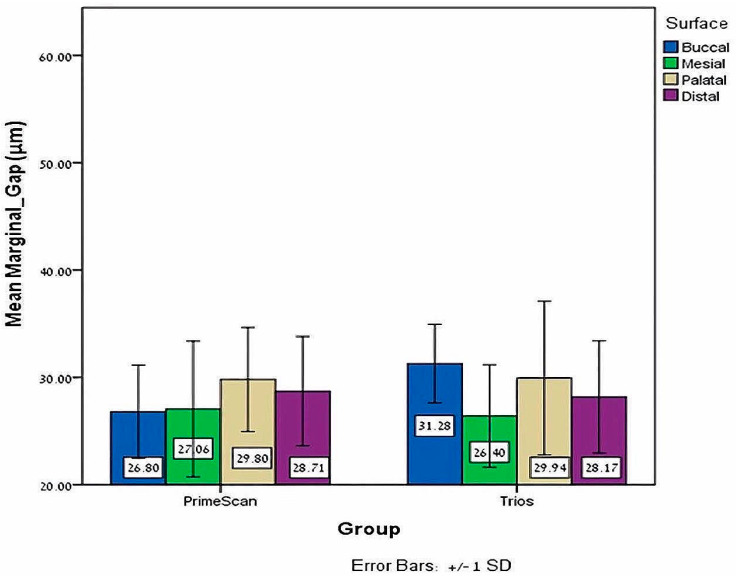
Mean and ±SD of the buccal, mesial, palatal and distal mean marginal gap (μm) with Temp-bond for both Primescan and Trios.

The independent *T* test found no significant marginal gap differences in the zirconia-reinforced lithium silicate crowns bonded with Temp-bond and scanned by Primescan compared to Trios, *p* = 0.401 (Table 1). Regarding the surfaces, using Primescan or Trios, no significant marginal gap differences were found on the palatal (*p* = 0.944), mesial (*p* = 0.715) and distal (*p* = 0.741) surfaces of crowns bonded with Temp-bond, with a significant difference only on the buccal surface (*p* = 0.001), where a larger marginal gap was found for crowns scanned by Trios (Table 2, Figure 4).

**Figure 5:**
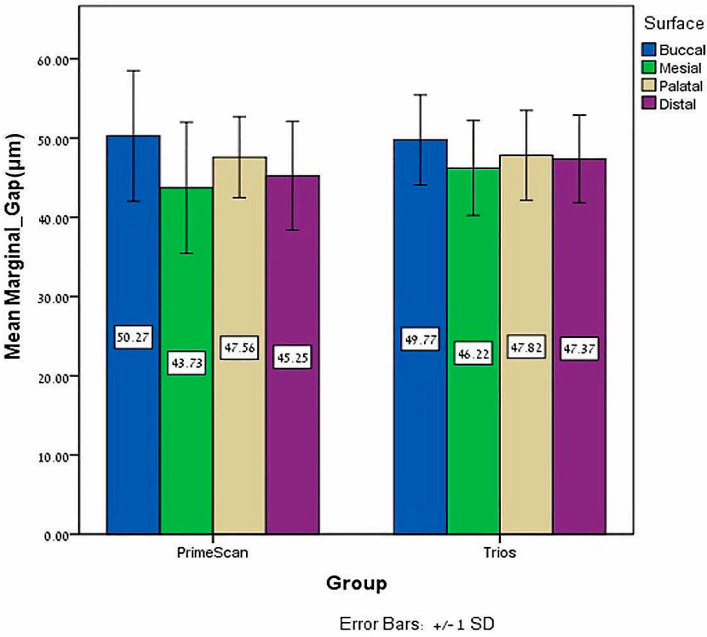
Mean and ±SD of the buccal, mesial, palatal and distal mean marginal gap (μm) with Gcem ONE for both Primescan and Trios.

The independent *T* test found no significant marginal gap differences of zirconia-reinforced lithium silicate crowns bonded with Gcem ONE and scanned by Trios compared to Primescan, *p* = 0.295 (Table 3). Regarding the surfaces, by using Primescan or Trios no significant marginal gap differences were found on the buccal (*p* = 0.824), mesial (*p* = 0.282) distal (*p* = 0.288) and palatal (*p* = 0.881) surfaces of crowns bonded with Gcem ONE (Table 4, Figure 5).

**Figure 6:**
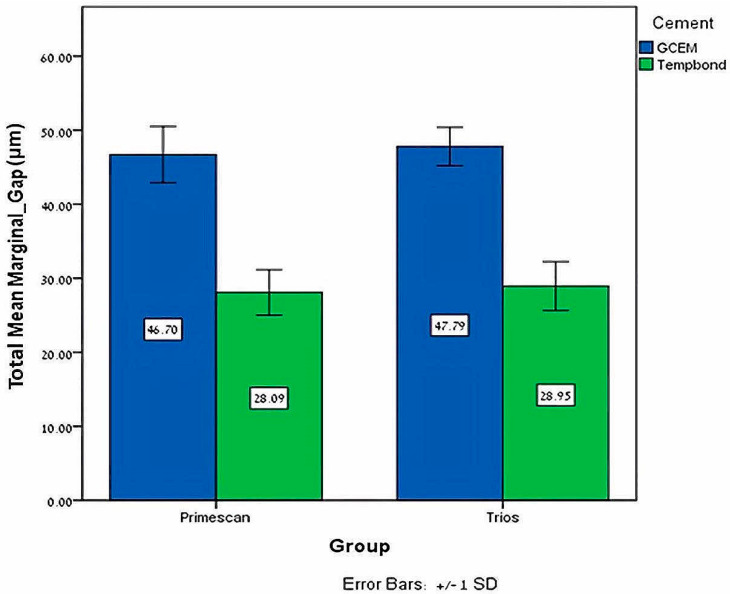
Mean and ±SD of the total mean marginal gap (μm) for Primescan and Trios with both cements.

When scanning with PrimeScan, the paired-sample *T* tests showed significantly larger marginal gaps with Gcem ONE compared to Temp-bond on all surfaces, buccal (*p* = 0.0005), mesial (*p* = 0.0005), palatal (*p* = 0.0005) and distal (*p* = 0.0005), and for the mean marginal gaps of all surfaces (*p* = 0.0005) (Figure 6).

When scanning with Trios, the paired-sample *T* tests revealed the same trend, in which significantly larger marginal gaps were found with Gcem ONE compared to Temp-bond at all surfaces, buccal (*p* = 0.0005), mesial (*p* = 0.0005), palatal (*p* = 0.0005) and distal (*p* = 0.0005), and for the mean marginal gaps of all surfaces (*p* = 0.0005) (Figure 6).

## 4. Discussion

In the current literature, there is not enough evidence regarding the effect of closed and open CAD/CAM systems on the marginal gaps of single monolithic crowns. In this study, we wanted to understand whether there is an effect of closed and open CAD/CAM systems on the marginal gaps of CELTRA^®^ DUO (Sirona Dentsply, Milford, DE, USA) single crowns, it was assumed that no significant differences would be found regarding the marginal gaps between the two systems and the two cements. The results only confirmed the first null hypothesis, as no differences were visible for both Temp-bond and Gcem ONE between the two systems, open and closed. Regarding the second null hypothesis, it had to be rejected as significant differences were found between cementation with Temp-bond and Gcem ONE. For both groups, the marginal gaps were within the limit of the clinically acceptable values (<120 µm) [15] suggested by a classic study and within the limit values (<80 µm) of a more current study regarding CAD/CAM systems [22].

Kricheldorf et al. compared the vertical marginal discrepancies of an open system versus a closed system by using titanium abutments with chamfer finish lines of 1.2 mm. They scanned 10 abutments for each group with CEREC Scanner 3D Bluecam (Sirona Dental Systems GmbH, Bensheim, Germany) for the closed system and Optimet Scanner DS6000 (Optimet, Jerusalem, Israel) for the open system. For both the closed and open system, they used different design and milling systems (closed system: four-axis versus open system: five-axis), milled fully felspathic porcelain single crowns, and used the replica technique with light addition silicone (Virtual, Ivoclar Vivadent, Schaan, Liechtenstein) to fix the crowns on the abutments. Eventually, they examined the gap with a stereomicroscope (Olympus SZX9, Tokyo, Japan) and found differences between the two groups, as the closed system group (23.75 µm ± 3.05) exhibited higher mean marginal discrepancies compared to the open system group (17.94 µm ± 4.77) [32]. In this study, plastic abutments were used and not titanium abutments, with shoulder finish lines and not chamfer. Primescan was compared to Trios 4, and ZLS crowns were grinded and not feldspathic, with the same four-axis machine, so only the CAD software was different. SEM with X250 magnification was used, not a stereomicroscope with X42 magnifications. Higher values of the marginal gap were found in the Temp-bond group for both the closed system (28.09 µm ± 3.06) and open system (28.94 µm ± 3.30), but without a significant difference between the groups.

In a previous study, the same abutment was used with the same restoration material as in this study, but they were used for both closed and open systems Omnicam (CEREC^®^ AC Omnicam; Dentsply Sirona, Milford, DE, USA). For the closed system, CEREC^®^ software was used, and for the open system, EXOCAD^®^ software was used. Both groups were grinded with a four-axis machine (CEREC inLab MC XL^®^; Dentsply Sirona, Milford, DE, USA), then bonded with self-adhesive resin cement (Rely X U-200; 3M ESPE) and evaluated under the same SEM with the same magnification of X250. The mean marginal gap for the closed system was 55.35 µm ± 4.83, and for the open system, 38.4 µm ± 4.35, with significant differences between the groups [33]. In this research, two different IOSs were used for the closed (Primescan) and open (Trios 4) systems. The design software was the same for both systems and the grinding machine (four-axis) as well. A different self-adhesive resin cement, Gcem ONE Auto mix (GCA; GC, Alsip, IL, USA), was used, and mean marginal gaps of 46.70 µm ± 3.80 for the closed system and 47.79 µm ± 2.59 for the open system were found, with no significant differences between the groups. In a previous study, it was concluded that the difference might have been due to data conversion when taking a file from a closed system and converting it to an STL file. In this study, for the open system, Trios 4 was used, and the STL file was received without conversion. This may explain why no differences were found between the two systems. The effect of conversion on digital files needs to be investigated more in the literature, as there are almost no articles regarding this issue.

A recent study by Akat et al. tried to find out whether the CAD software has an impact on the marginal and internal fit of full ceramic crowns; they used typodont maxillary first molar teeth with a rounded shoulder finish line. Three groups of 11 teeth in each were scanned, all by Trios 3 (3Shape, Copenhagen, Denmark), and the STL files were used by three different CAD systems (CEREC (inLab 15.1), KaVo (multicad. PC_V4.0.3) and Planmeca (Romexis PlanCad Easy5.9.2.09). The radial spacer was defined as 80 µm and the crowns were milled from feldspathic porcelain (62790; VITA Zahnfabric, Bad Säckingen, Germany) by a five-axis machine. The crowns were cemented with paraffin, and measurements with a two-dimentional (2D) method were taken. They found significant differences in the marginal gap between the Planmeca group (151.9 µm ± 93.1) compared to the other two groups, CEREC (25.6 µm ± 47.3) and KaVo (31.3 µm ± 65.5). In this study, although different IOS and CAD systems (CEREC^®^ SW 5.2.4; Dentsply Sirona and EXOCAD^®^ DentalCAD 2.2 Vallenta, Darmstadt, Germany) were used, no differences were found between the two systems, and the values for the Temp-bond group were similar (28.09 µm ± 3.06 and 28.94 µm ± 3.30) to those of the CEREC and KaVo CAD systems [34].

In this study, no differences were found between the open system and closed system regarding the marginal gap; it can be assumed that as technology advances, the differences will diminish. Two current advanced IOSs were used and compared, while the same four-axis machine was used for both systems, and this might be the reason why no significant differences were found. The CAD systems are also improving as time passes by, and this might be another reason explaining this issue.

When investigating the differences between the two groups (Temp-bond versus Gcem ONE), it was found that the self-adhesive resin cement caused an elevation of 18–20 µm compared to the Temp-bond group. A study by Pilo et al. which examined the effect of cementation with self-adhesive resin cement on the marginal gap of monolithic zirconia single crowns showed that before cementation, the mean marginal gap was 34.83 µm, and after cementation, it was 72 µm, a difference of 37 µm [35]. The difference between the two studies (18–20 µm versus 37 µm) can be explained by the differences between the self-adhesive resin cements, and it is known that the viscosity, which is affected by the filler content and geometry, can affect the positive seat of the crown, and hence increase the marginal gap [36].

More self-adhesive and adhesive resin cements should be tested in future studies to understand what their effect on the marginal gaps of different materials and types of restorations is.

Regarding the overall results, it is important to mention that the measuring technique also influences the results [37,38]. In this study, only the 2D direct technique was used with SEM, which, among the 2D methods is the most used in the literature [38].

There are some limitations to this study, such as the fact that it was an in-vitro study, one type of preparation was used, plastic teeth were used, one type of material was used, one type of self-adhesive resin cement was used, one type of measuring technique (direct) was used and only two types of IOSs were used.

Further studies are required to understand the influence of closed versus open systems on the marginal gap, especially to understand what the true influence of file conversion on the accuracy of digital dentistry (implants and teeth) is and if different CAD systems may affect the marginal gap.

## 5. Conclusions

Despite the limitations of this in vitro study, the following can be suggested:

1. In everyday dentistry, both closed systems and open systems can be used to achieve an acceptable marginal gap (<120 µm) for ZLS CELTRA^®^ DUO single crowns.
2. No significant difference was found regarding the marginal gap when comparing ZLS CELTRA^®^ DUO single crowns produced by closed systems or open systems and cemented with Gcem One self-adhesive resin cement.
3. There was a significant difference between cementation with Temp-bond and Gcem One self-adhesive resin cement regarding the marginal gap (*p* < 0.0005).

## Figures and Tables

**Table 1:** Mean, ±SD, confidence interval (upper/lower limit), minimum and maximum of the total mean marginal gap (μm) of zirconia-reinforced lithium silicate crowns bonded with Temp-bond and scanned by Trios or Primescan (α = 0.0005).

Total Mean Marginal Gap Temp-Bond (μm)	Mean ±SD	CI—Upper/ Lower Limit	Min Max
PrimeScan	28.09 ±3.06	29.52 26.65	20.75 32.88
Trios	28.94 ±3.30	30.49 27.39	24.54 34.79

**Table 2:** Mean, ±SD, confidence interval (upper/lower limit), minimum and maximum of the distal, mesial, palatal and buccal mean marginal gaps (μm) of zirconia-reinforced lithium silicate crowns bonded with Temp-bond and scanned by Trios or Primescan (α = 0.0005).

	Buccal Surface			Mesial Surface			Palatal Surface			Distal Surface		
Mean Marginal Gap Temp-Bond (μm)	Mean ±SD	CI—Upper/ Lower Limit	Min Max	Mean ±SD	CI—Upper/ Lower Limit	Min Max	Mean ±SD	CI—Upper/ Lower Limit	Min Max	Mean ±SD	CI—Upper/ Lower Limit	Min Max
Primescan	26.79 ±4.35	28.83 24.76	21.20 34.84	27.05 ±6.32	30.01 24.09	18.39 39.39	29.80 ±4.84	32.07 27.53	21.27 38.31	28.70 ±5.08	31.08 26.33	18.17 35.42
Trios	31.27 ±3.66	32.99 29.56	24.10 37.73	26.40 ±4.77	28.63 24.16	19.16 36.93	29.94 ±7.15	33.29 26.59	17.97 46.05	28.16 ±5.22	30.61 25.71	19.08 42.05

**Table 3:** Mean, ±SD, confidence interval (upper/lower limit), minimum and maximum of the total mean marginal gap (μm) of zirconia-reinforced lithium silicate crowns bonded with Gcem and scanned by Trios or Primescan (α = 0.0005).

Total Mean Marginal Gap Gcem ONE (μm)	Mean ±SD	CI—Upper/ Lower Limit	Min Max
PrimeScan	46.70 ±3.80	48.48 44.92	38.42 52.47
Trios	47.79 ±2.59	49.00 46.57	42.67 52.72

**Table 4:** Mean, ±SD, confidence interval (upper/lower limit), minimum and maximum of the distal, mesial, palatal and buccal mean marginal gap (μm) of zirconia-reinforced lithium silicate crowns bonded with Gcem ONE and scanned by Trios or Primescan (α = 0.0005).

	Buccal Surface			Mesial Surface			Palatal Surface			Distal Surface		
Mean Marginal Gap Gcem ONE (μm)	Mean ±SD	CI—Upper/ Lower Limit	Min Max	Mean ±SD	CI—Upper/ Lower Limit	Min Max	Mean ±SD	CI—Upper/ Lower Limit	Min Max	Mean ±SD	CI—Upper/ Lower Limit	Min Max
Primescan	50.26 ±8.22	54.11 46.41	33.07 62.67	43.72 ±8.27	47.59 39.85	27.72 57.44	47.56 ±5.09	49.94 45.17	35.90 59.00	45.24 ±6.86	48.45 42.03	35.54 59.16
Trios	49.76 ±5.67	52.42 47.11	32.28 57.50	46.21 ±5.97	49.01 43.42	34.49 56.14	47.82 ±5.66	50.47 45.16	34.65 56.60	47.36 ±5.52	49.94 44.78	36.35 54.95

## Data Availability

The data presented in this study are available upon reasonable request from the corresponding author.
